# Differences in Dynamics of Lung Computed Tomography Patterns between Survivors and Deceased Adult Patients with COVID-19

**DOI:** 10.3390/diagnostics11101937

**Published:** 2021-10-19

**Authors:** Gevorg B. Akopyan, Alexander B. Berdalin, Ilya L. Gubskiy, Vladimir G. Lelyuk

**Affiliations:** Radiology and Clinical Physiology Research Center, Federal State Budgetary Institution Federal Center of Brain Research and Neurotechnologies of the Federal Medical Biological Agency, 117513 Moscow, Russia; berdalin.a@fccps.ru (A.B.B.); gubskiy.i@fccps.ru (I.L.G.); vglelyuk@fccps.ru (V.G.L.)

**Keywords:** COVID-19, SARS-CoV-2, computed tomography, patterns, dynamics, prognostic factor, ground glass opacity (GGO), reticular changes (RC), crazy paving (CP)

## Abstract

This study’s aim was to investigate CT (computed tomography) pattern dynamics differences within surviving and deceased adult patients with COVID-19, revealing new prognostic factors and reproducing already known data with our patients’ cohort: 635 hospitalized patients (55.3% of them were men, 44.7%—women), of which 87.3% had a positive result of RT-PCR (reverse transcription-polymerase chain reaction) at admission. The number of deaths was 53 people (69.8% of them were men and 30.2% were women). In total, more than 1500 CT examinations were performed on patients, using a GE Optima CT 660 computed tomography (General Electric Healthcare, Chicago, IL, USA). The study was performed at hospital admission, the frequency of repetitive scans further varied based on clinical need. The interpretation of the imaging data was carried out by 11 radiologists with filling in individual registration cards that take into account the scale of the lesion, the location, contours, and shape of the foci, the dominating types of changes, as well as the presence of additional findings and the dynamics of the process—a total of 45 parameters. Statistical analysis was performed using the software packages SPSS Statistics version 23.0 (IBM, Armonk, NY, USA) and R software version 3.3.2. For comparisons in pattern dynamics across hospitalization we used repeated measures general linear model with outcome and disease phase as factors. The crazy paving pattern, which is more common and has a greater contribution to the overall CT picture in different phases of the disease in deceased patients, has isolated prognostic significance and is probably a reflection of faster dynamics of the process with a long phase of progression of pulmonary parenchyma damage with an identical trend of changes in the scale of the lesion (as recovered) in this group of patients. Already known data on typical pulmonological CT manifestations of infection, frequency of occurrence, and the prognostic significance of the scale of the lesion were reproduced, new differences in the dynamics of the process between recovered and deceased adult patients were also found that may have prognostic significance and can be reflected in clinical practice.

## 1. Introduction

Coronavirus disease 2019 is a new epidemic infectious disease characterized by relatively high contagiousness for humans, the probability of developing life-threatening complications in the form of acute respiratory distress syndrome, acute respiratory and multiple organ failure, the causative agent of which is the enveloped zoonotic RNA virus SARS-CoV-2. The virus belongs to the family Coronaviridae, the genus Betacoronavirus (as well as the previously known SARS-CoV viruses of 2002–2004 and MERS-CoV 2013–2015, which differs by significantly lower fatality rate), transmitted by airborne droplets, airborne dust, and contact ways and affects mainly ACE2R-positive cells (angiotensin-converting enzyme 2 receptor) due to its S-protein and influencing on it membrane-associated protease serine (TMPRSS2) [1,2,3]. ACE2R-positive cells are widely represented in the human body. The main target cells for the virus are the cells of the nasal–oral–pharyngeal and respiratory tract mucosa (including alveoli), as well as endothelial cells (which may cause the development of hypercoagulation syndrome and thromboembolism) [4,5]. Clinical manifestations of infection vary from mild or moderate (clinical phenotypes L, 70–80% of all cases, without signs of viral pneumonia or hypoxia) to severe or extremely severe (clinical phenotype H or ARDS, 20% of all cases) [6,7]. The whole spectrum of symptoms is wide and non-specific. The incubation period is 5–7 days on average (from 1 to 14 days, in 12.5% of cases-more than 14 days) [8,9]. The patient can secrete the virus 2 days before the onset of symptoms and up to 10 days after the onset of symptoms, in some cases (depending on the severity of the clinical course)—up to 20 days [10,11,12]. Mortality among patients is 2–3.6% [13], the probability of death is higher among men, increases in the presence or decompensation of concomitant pathology (diabetes mellitus, arterial hypertension, diseases of the cardiovascular system, etc.), as well as in people over the age of 60 years [14,15,16,17,18,19,20]. The epidemic currently covers almost the whole world, and, according to WHO Coronavirus Dashboard, for the entire period from December 2019 (first official reports of cases of atypical pneumonia in Wuhan city, Hubei province, China) up until 5 October 2021 there have been more than 234,809,000 cases (including Russia—more than 7,612,000), and more than 4,800,000 deaths (in Russia—more than 210,800). At the time of writing, no specific etiotropic therapy has been described and, despite quarantine measures, and different mRNA (mRNABNT162b2—Pfizer-BioNtech, Manhattan, NY, USA; mRNA-1273—Moderna, Cambridge, MA, USA) or vector-based (JNJ-78436735—Johnson & Johnson, New Brunswick, NJ, USA; ChAdOx1-S—AstraZeneca, Cambridge, England; Gam-COVID-Vac—Gamaleya NRC of Epidemiology and Microbiology, Moscow, Russia) vaccines, developed and applied on various scales, the number of cases in the world continues to increase by 257,100 daily (in the Russian Federation—25,700) [21]. It is worth noting that concern remains because, according to data of Brogna et al. and several authors, the rapid spread of new virus genetic variants (e.g., Delta) and the absence of an effective immune response leads to an increase in the number of infections in fully vaccinated people [22]. Besides, the actual number of infections may be significantly higher due to insufficiently effective and widespread diagnostics.

The diagnosis for COVID-19 is mainly confirmed by reverse transcription-polymerase chain reaction (PCR). Despite the presence of some disadvantages of this method, the majority of authors do not recommend using CT as a substitute for it [23]. Nevertheless, the method helps to exclude the presence of alternative or additional causes of the occurrence and development of pulmonological symptoms, as well as possible complications. It also assists in clinical monitoring and allows us to improve accuracy by integrating open-source or commercial semiautomatic or automatic and artificial intelligence software for objective recognition, categorization, and quantification of lung disease [24].

In connection with the threatening epidemiological situation, many difficulties arose associated with access to clinics and hospitals, and obtaining standard and specialized diagnostic and medical care, which led to the need of reshaping patient routing, algorithms of performing manipulations [25]. Many federal guidelines and local solutions have been developed. The main goals were to reduce the risk of infection of medical personnel and the spread of infection among patients. In addition, several medical and Federal research centers located in Moscow were redesigned to provide medical assistance to persons with novel coronavirus infection. In the period from April to June 2020 Radiology and clinical physiology research center of Federal State Budgetary Institution “Federal Center of Brain Research and Neurotechnologies” of the Federal Medical Biological Agency (RCPRC of FCBRN FMBA) created a large database of clinical, laboratory, and instrumental data.

### Purpose of the Article

This study aimed to investigate computed tomography patterns dynamics differences within surviving and deceased adult patients with COVID-19 revealing new prognostic factors with our patients’ cohort.

## 2. Materials and Methods

### 2.1. Patients

635 hospitalized patients (55.3% of them were men%—351 people, 44.7%—women%—284 people), of which 87.3% had a positive result of RT-PCR at admission. In total, more than 1500 CT examinations were performed on patients.

The average age of the examined patients was 59 years (from 20 to 100 years, the standard deviation is 15 years). The number of deaths was 53 people (8.3% of those included in the analysis): 69.8% of them were men (37 people) and 30.2% were women (16 people). In 28 cases out of 53 (52.8%), the deceased patients developed ARD syndrome.

### 2.2. Methods

CT examinations of the chest organs were performed on the maximum inspiration in the supine position with raised arms, using a GE Optima CT 660 computed tomography (General Electric Healthcare, Chicago, IL, USA). The voltage value on the tube in most cases was 120 kV, with automatic current modulation of 150–350 mA (depending on the constitution and weight of the patient), with collimation of the slice thickness of 0.625 mm and a hard convolution kernel.

The study was performed at hospital admission, the frequency of repetitive scans further varied based on clinical need (worsening of respiratory status, disease progression, complications diagnosis, or the evaluation of the dynamics of the changes) and by the requirements of the local regulatory guidance documents on the management of patients with COVID-19. As a summary, there were from 1 to 7 scans for every patient. The time intervals between repeated examinations were not the same in different patients. On average, a hospitalized patient spent from 18 to 27 days in the hospital (median—15 days). The results of CT studies are described depending on the time since the development of clinical symptoms.

The interpretation of the imaging data was carried out by 11 radiologists with filling in individual registration cards that take into account the scale of the lesion (segmentally, according to a point system from 0 to 2) [26], the location, contours, and shape of the foci, the dominating types of changes, as well as the presence of additional findings and the dynamics of the process—a total of 45 parameters.

Impact of different lung damage patterns was assessed with such method: scores of all patterns were summed and then the percentage for each pattern in the summed score of all patterns was calculated.

### 2.3. Statistical Analysis

Statistical analysis was performed using the software packages SPSS Statistics version 23.0 (IBM, Armonk, NY, USA) and R software version 3.3.2. The null hypothesis was rejected at a significance level of *p* < 0.05. For the description of scale variables, the arithmetic means and standard deviation or median and quartiles were used (if the distribution of the variable did not match the normal one), for nominal variables, the frequency and proportion (in percent) were used. The distribution of scale variables was assessed by frequency histogram evaluation. For nominal dependent variables, frequency comparison between categories of independent (grouping) variables were performed using the criterion χ2 Pearson or the exact Fischer criterion. For scale dependent variables, analysis was made using ANOVA followed by pairwise comparisons using the Dunnett method or (if the distribution of the variable did not match the normal one)—Kruskal–Wallis criteria with pairwise comparisons by the Mann–Whitney criterion (using Bonferroni multiplicity adjustment). For comparisons in pattern dynamics across hospitalization, we used a repeated measures general linear model with outcome and disease phase as factors.

## 3. Results

### 3.1. Clinical Information

Fifty-six patients reported smoking (8.8%). Arterial hypertension was detected in 318 patients (50.1%), myocardial infarction—in 50 patients (7.9%), 25 patients had a stroke before (3.9%), COPD was found in 41 patients (6.5%), 18 patients suffered bronchial asthma (2.8%), 32 patients were admitted with known cancer after treatment (5%), 97 patients (15.3%) suffered from well-controlled diabetes mellitus. The main results of our patients’ epidemiological risk factors analysis are shown in the Table 1.

### 3.2. CT Manifestations of Coronavirus Infection

The CT patterns described in the research papers of other authors [28] and histological [29,30] changes in patients with COVID-19 are similar to those in the previously described SARS-CoV-1 and MERS-CoV and overlap with the manifestations of other viral infections (typical and atypical).

The so-called ‘basic’ typical manifestations of coronavirus infection according to many (and there are currently more than 65) authors include [13,31,32,33,34,35,36,37,38]: the presence of numerous bilateral multilobar, mostly rounded sections GGO and consolidations (different length—focal or drain), as well as halo or reverse halo pattern of increasing density of the lung tissue; linear septal thickening or bands—a pattern of reticular changes; as well as the pattern of CP, which is a combination of the first two patterns. Some authors also describe perilobular consolidations. Secondary typical manifestations include microcavitation (air bubble sign), cylindrical bronchiolectasis, subpleural arcuate lines, and pleuroparenchymal bands, dilation of the terminal parts of the vessels, areas of local thickening, and traction of the pleura. Hydrothorax, lymphadenopathy, and pulmonary nodules are described in a small number of cases.

In our work, we focused exclusively on the study of the main CT manifestations of coronavirus infection and their characteristics.

#### 3.2.1. Ground-Glass Opacity

**Figure 1:**
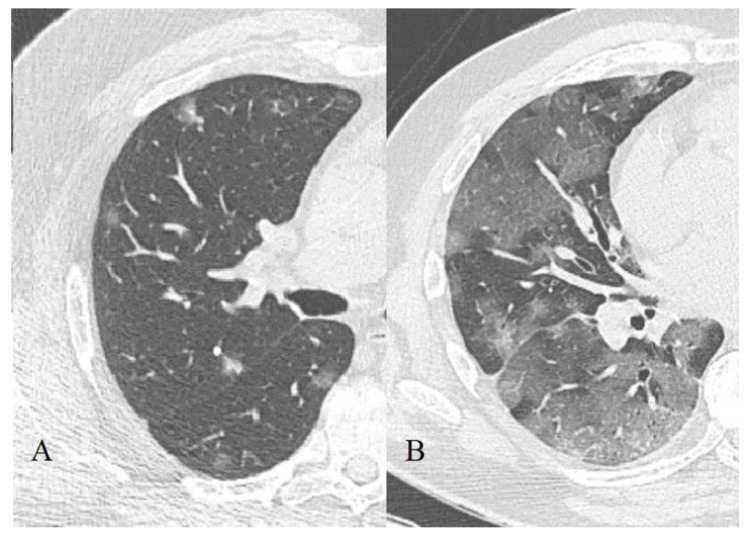
Ground glass opacity, focal (**A**) and confluent (**B**) form.

Area of the lung parenchyma with moderately increased density with the preservation of volume and the possibility of anatomical components of the pulmonary lobule differentiation (walls of bronchioles, venules, and arterioles, Figure 1). Often, the contours of these sections are fuzzy. The symptom is based on the process of partial replacement of air with pathological contents (fluid, cells, fibrosis) or interstitial compaction, or partial collapse of the alveoli, an increase in capillary blood flow; or a combination of all of these changes [39].

**Figure 2:**
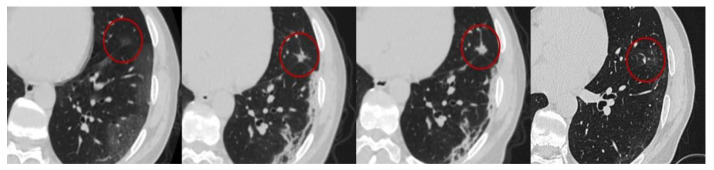
The red circle marks the area of ground-glass opacity, which transforms into consolidation (nodular pattern) area and vice versa during repetitive studies.

Such changes can progress into areas of consolidation (Figure 2) and (or) CP (see below), as well as remain residual during their regression (since air is still partially replaced by other contents during reparative processes). This is supported by the COVID-19 patients autopsy data in which the same histological changes are found in the areas of GGO and consolidations, moreover, of a very wide spectrum [40].

According to our data, GGO is the most common finding, it was found on CT images in 87% of cases in recovered persons and 86.2%-in the dead. The recovered patients also showed a greater contribution of this pattern to the overall CT picture (on average, 37 ± 26% versus 31 ± 18.7%).

A symptom of dilation of the terminal parts of the vessels and traction bronchiolectasis is often superimposed with this type of change [41].

#### 3.2.2. Consolidation

**Figure 3:**
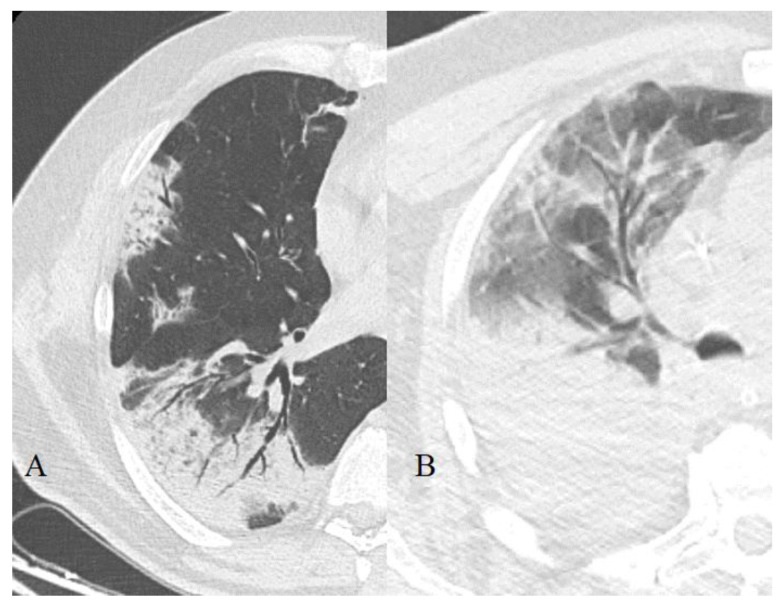
Consolidation with “air bronchogram” sign (**A**) or in a patient with Acute respiratory distress syndrome (ARDS) (**B**).

An area of the lung parenchyma intensively increased density with the preservation of volume, but inside this pattern the anatomical components of the pulmonary lobule are not distinguishable (Figure 3). This displays the process of complete replacement of the alveolar air with pathological contents [39].

**Figure 4:**
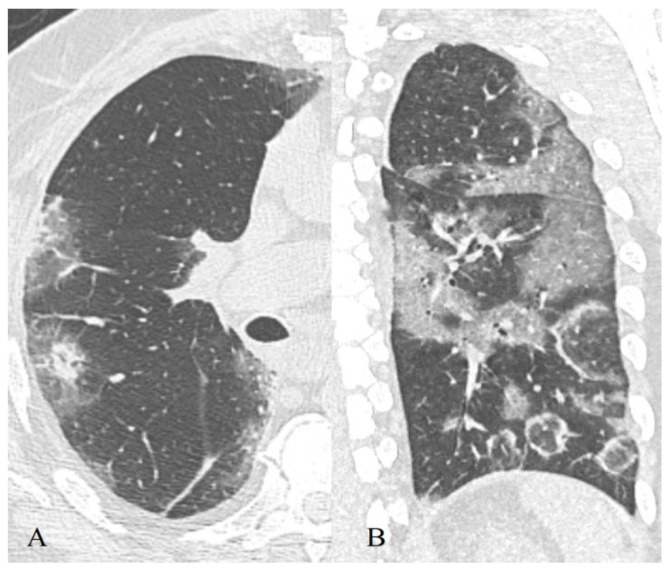
Consolidation types: halo phenomena (**A**) and reverse halo (**B**) with perilobular bands.

Consolidation zones can be homogeneous, as well as heterogeneous (in the case of progression or, conversely, regression, they may contain areas of different densities—for example, GGO or normal airiness, Figure 4). Often, these changes are superimposed with the symptom of an “air bronchogram”.

Consolidations according to our data occurred in 67.4% of cases in those who recovered and in 80.2%—in those who died. Additionally, we had a greater contribution of this pattern to the overall picture (on average, 27 ± 23% versus 22 ± 21.5%).

#### 3.2.3. “Crazy Paving” Pattern

**Figure 5:**
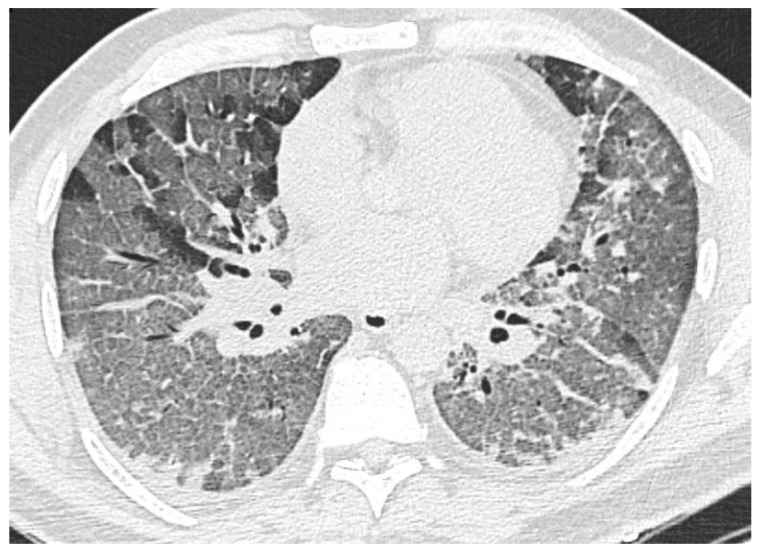
Crazy paving pattern.

The phenomenon of “CP” corresponds to intra-and inter-lobular septal thickening on top of “GGO” changes, which is a reflection of pathological changes in both compartments: alveolar and interstitial [39] (Figure 5).

Often, such areas are clearly separated from the intact lung tissue and are intermediate between “GGO” changes and consolidation. The phenomenon of “CP” according to our data was registered in 42.3% of studies in those who recovered and in 72.2%—in those who died. Additionally, the contribution of this pattern to the overall picture was higher than that of the survivors (on average, 23 ± 20% versus 11 ± 17.2%).

#### 3.2.4. Reticular Interstitial Pattern, Pleuroparenchymal Bands

**Figure 6:**
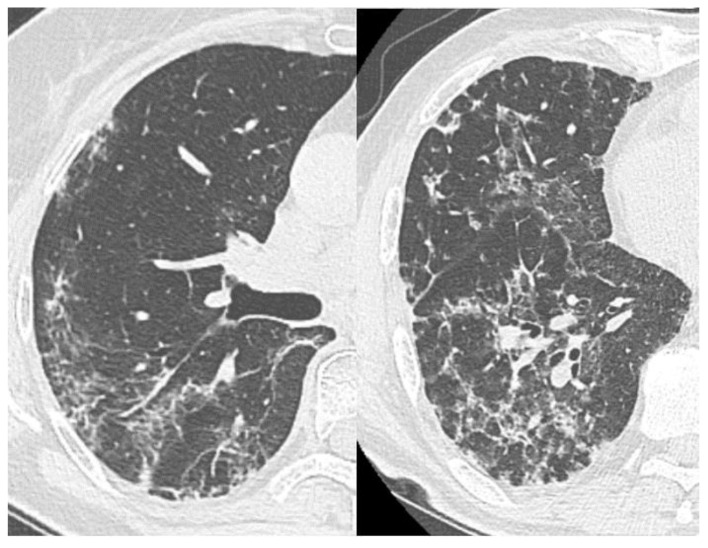
Reticular interstitial pattern.

The reticular interstitial pattern suggests the presence of multiple small linear bands, in a complex resembling a network, which is a reflection of the pulmonary lobule interstitium pathology [39] (Figure 6).

**Figure 7:**
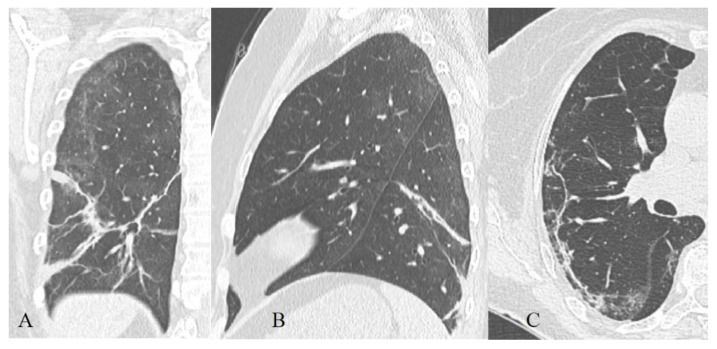
Linear (**A**,**B**) and curvilinear (**C**) pleuroparenchymal bands.

Pleuroparenchymal bands are linear areas of consolidation, 3–5 mm thick, extending to the pleura (which, in turn, can be thickened and with traction changes at the contact point) [39]; they correspond to pleuroparenchymal fibrosis and can be associated with other disorders of the architecture of the lung tissue (Figure 7).

RC and pleuroparenchymal bands were registered in 61% and 58% of cases in recovered patients, respectively, and in 59% and 47%—in the dead. The recovered patients had a greater contribution of these changes to the overall CT picture (on average, 16.8 ± 17% vs. 11.3 ± 11.1% and 13 ± 14.1% vs. 7.6 ± 10%).

#### 3.2.5. Localization, Shape, and Volume of the Lesion

According to our data, the isolated peripheral location was found in 70.5% of recovered patients, equally peripheral and perihilar in 27.5%, an isolated perihilar location of pathological areas in 2%. In the deceased, values were 40%, 59.2%, and 0.8%, respectively. There was also a frequency increase of approximately equally peripheral and basal location of the affected areas with the course of the disease (33.3% > 45% > 59% > 70%, *p* < 0.05). Similar dynamics were observed in the group of recovered patients, but to a lesser extent, which is quite natural, given the increase in severity with the involvement of more and more areas of the pulmonary parenchyma.

The shape of the lesions in both the recovered (in 85%) and the dead (in 97.5% of cases) was irregular. The contours in both cases were mostly indistinct (72–75%).

In those who recovered, the severity of the lesion of the pulmonary parenchyma was lower in the sum of points than in the deceased: 17.3 ± 8 versus 28.5 ± 10.5.

#### 3.2.6. Hydrothorax, Hydropericardium, Lymphadenopathy

Fluid in the pleural cavity and lymphadenopathy were more often observed in deceased patients—30% and 14% versus 11% and 2% in recovered patients, respectively. At the same time, there was an increase in the frequency of occurrence of the indicated changes with the course of the disease (*p* < 0.05).

However, it should be noted that the threshold for identifying lymphadenopathy is very variable. Usually, markers are the total number of lymph nodes and their size.

### 3.3. Dynamics of CT Changes over Time

**Figure 8:**
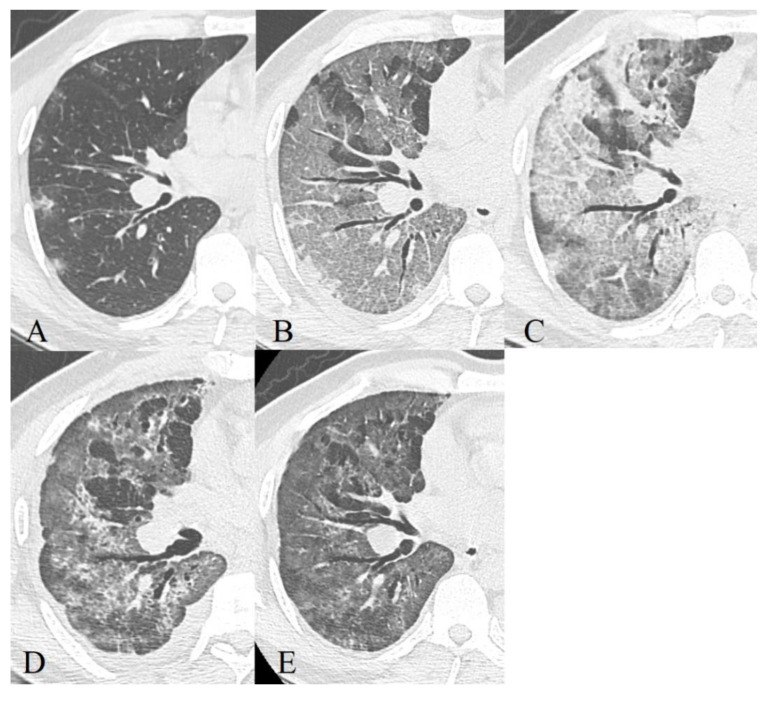
44-year-old patient, m, 11.04 symptom onset, 17.04 PCR+, AT к Il-6 used for therapy, images obtained 16.04 (**A**), 21.04 (**B**), 24.04 (**C**), 13.05 (**D**), 21.05 (**E**), recovered.

**Figure 9:**
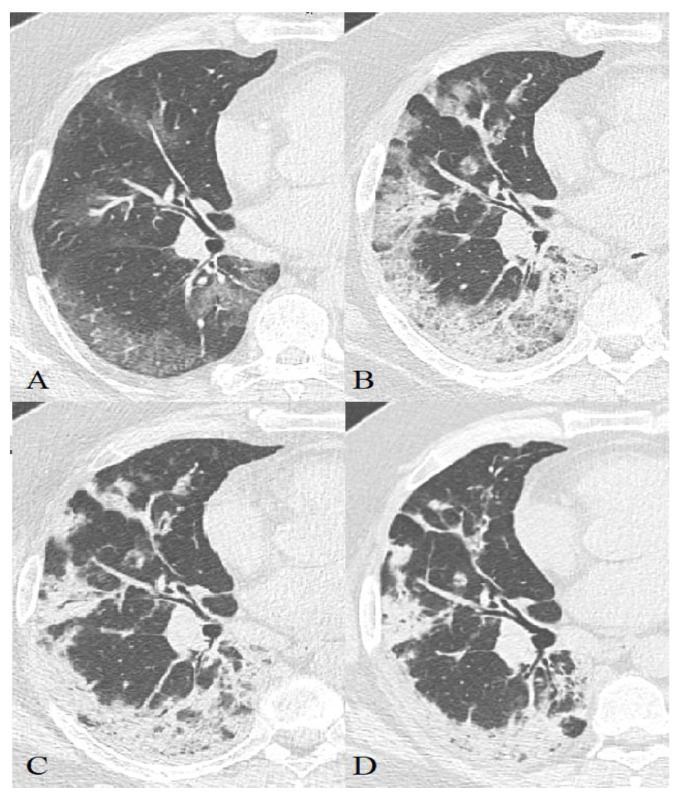
57-year-old patient, m, 14.04 symptom onset, 21.04 PCR+, additional oxygenation for therapy, images obtained 21.04 (**A**), 27.04 (**B**), 30.04 (**C**), 07.05 (**D**), recovered.

The temporal dynamics of all CT changes in COVID-19 (Figure 8 and Figure 9) with a high degree of a probability corresponds to that in other inflammatory lungs changes and does not correlate with histopathological dynamics, and also lags behind the clinical picture [40].

When describing the features of CT symptoms depending on the time that has elapsed since the onset of clinical symptoms, most researchers, including us, cite the work of F. Pan et al. [42], in which four radiological phases of the regular course of the process were identified, which Zhou et al. [43] characterized:

1. Early or initial (0–4 days): normal CT scan or the presence of areas of the GGO type;
2. Progressive (5–8 days): an increase in the volume of the GGO type lesions (the frequency of isolated registration is ~40%) and the appearance of CP zones; a combination of GGO and consolidations (in ~43%), GGO and RC (in ~58%); the presence of only consolidations (in ~12%) [43];
3. Peak (9–13 days): a significant decrease in the frequency of isolated GGO type changes with a predominance of a combination of GGO and RC (the combination of GGO and consolidations occurred in 30% of cases and already showed a downward trend, in 13% of cases there was a development of pleural effusion, compared to 2.3% during the previous phase) [43];
4. Resolution (>14 days): an increase in the volume of the air pulmonary parenchyma with the appearance and predominant contribution of RC, parenchymal bands, and subpleural lines, a reversible halo (signs of organizing pneumonia). At the same time, there is a decrease in the contribution of the combination of GGO and consolidations to the overall CT picture (~9%), with the beginning of a downward trend in the contribution of the combination of GGO and RC (~66%). The contribution of the isolated GGO pattern remained unchanged in comparison with the previous phase (~22%) [43] since consolidation was probably resolved into GGO.

Our results (listed below in Table 2) to some extent reproduced the aforementioned data.

The time intervals we used were shorter, but this turned out to be excessive since the rate of the dynamics of changes in the CT pattern was not fast enough, so in the final analysis we used the model of F. Pan et al., disease radiologic phases in our patients characterized.

1. Early or initial (0–4 days): GGO type changes dominated in both cohorts, but almost 67% of deceased CT studies had CP pattern in this phase of disease, almost half of studies demonstrated mixed GGO-consolidation changes;
2. Progressive (5–8 days): an increase in the volume of the GGO, appearance and increase CP (56% of recovered patient’s studies, 73%—deceased), consolidations (57% of recovered patient’s studies, 87%—deceased) type lesions in both cohorts, RC presented in half of the studies;
3. Peak (9–13 days): predominance of a combination of GGO (87%), consolidations (67%), and RC (54%) in recovered patients and the combination of CP (87%), consolidations (77%) and RC (65%)—in deceased. RC demonstrated ascending trend in both cohorts, consolidations—in recovered patients, CP—in deceased, the contribution of GGO decreased in both;
4. Resolution (>14 days): predominant contribution of consolidations and RC in both cohorts, at the same time there is a decrease in the contribution of GGO, but CP contribution decreased more slowly in deceased than in recovered patients.

#### 3.3.1. Severity of Lung Damage

According to our data, there is a statistically significant difference between the groups of patients with different outcomes in the average score values obtained as a result of the pulmonary parenchyma lesion assessment during CT in all phases of the disease. The average difference was ~12.5 points (*p* < 0.005)

There were significant changes in the average score values depending on the phase of the process (*p* = 0.014): the maximum difference was observed between the early (0–4 days) and the progression phase (5–8 days) and amounted to ~7.7 points in a pairwise comparison, after the peak stage there was a tendency to decrease the severity of damage (*p* < 0.005). At the same time, the dynamics of the process in both groups were similar

#### 3.3.2. Contribution of the ‘GGO’ Pattern to CT Picture in the Affected Areas

**Figure 10:**
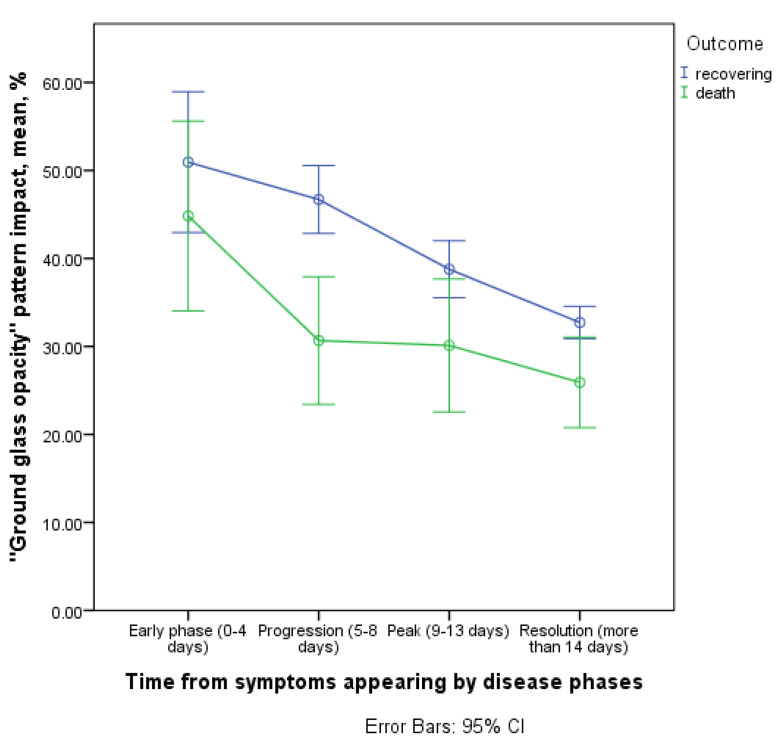
Contribution of the GGO pattern to CT picture in the affected areas.

There was a statistically significant difference in the ‘GGO’ pattern contribution to the overall CT picture dynamics in affected areas between the two outcomes (interaction of the time and outcome factor, *p* = 0.037, Figure 10).

The gap between the ‘GGO’ pattern contribution between those who recovered and those who died was observed in the progression phase (5–8 days) (46.7% [43; 50.4–95% CI] and 30.7% [20.1; 41.2–95% CI], respectively), which probably reflects a greater contribution of the ‘CP’ pattern in the group of patients who died subsequently.

The ‘GGO’ contribution was generally greater in patients who subsequently recovered (*p* = 0.017 for isolated outcome factor).

#### 3.3.3. Contribution of the ‘Crazy Paving’ Pattern to CT Picture in the Affected Areas

**Figure 11:**
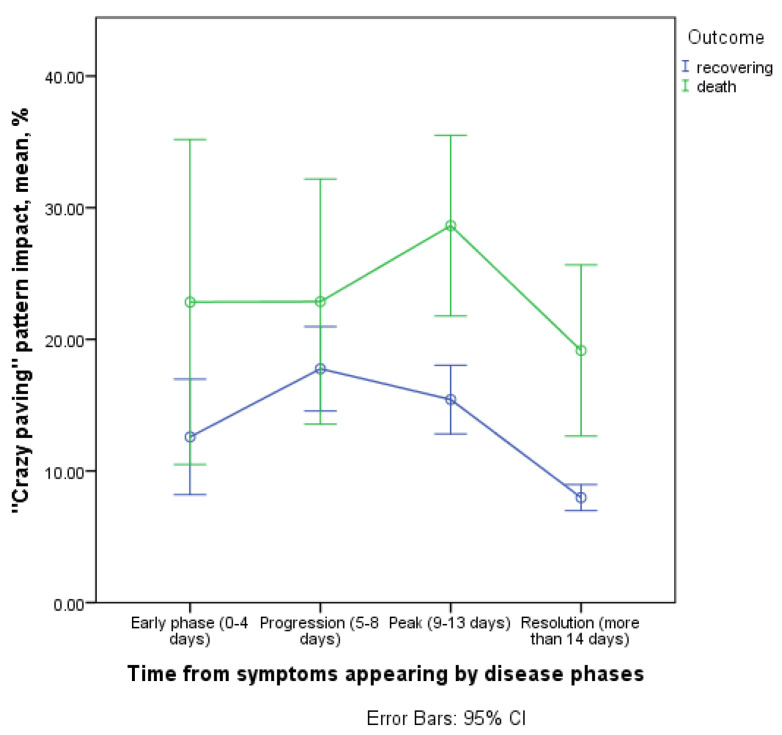
Contribution of the ‘crazy paving’ pattern to CT picture in the affected areas.

We found statistically significant differences in the contribution of ‘crazy pavement’ to total lung damage dynamics by the course of disease phases (interaction of the time and outcome factor, *p* = 0.007, Figure 11).

The dynamics of ‘crazy pavement’ contribution between the outcomes were generally similar in the time interval from 9 days and later (in the peak and the resolution phases) a gradual decrease in this pattern contribution was observed. However, in the initial phases of the process, there was a more pronounced and delayed increase in the ‘crazy pavement’ contribution in deceased patients, reaching a maximum in the peak phase of the disease (9–13 days). In recovered patients, the maximum of ‘crazy pavement’ contribution was observed in the progression phase (5–8 days). In this phase, the average contribution of the discussed pattern was 15.4% (13.3; 17.5–95% CI) and 28.7% (22.5; 34.8–95% CI) for recovered and deceased patients, respectively.

In addition, the ‘crazy pavement’ contribution was generally greater in patients who subsequently died (*p* = 0.008 for isolated outcome factor; mean difference was 10% approximately).

#### 3.3.4. Contribution of the Consolidation Pattern to CT Picture in the Affected Areas

**Figure 12:**
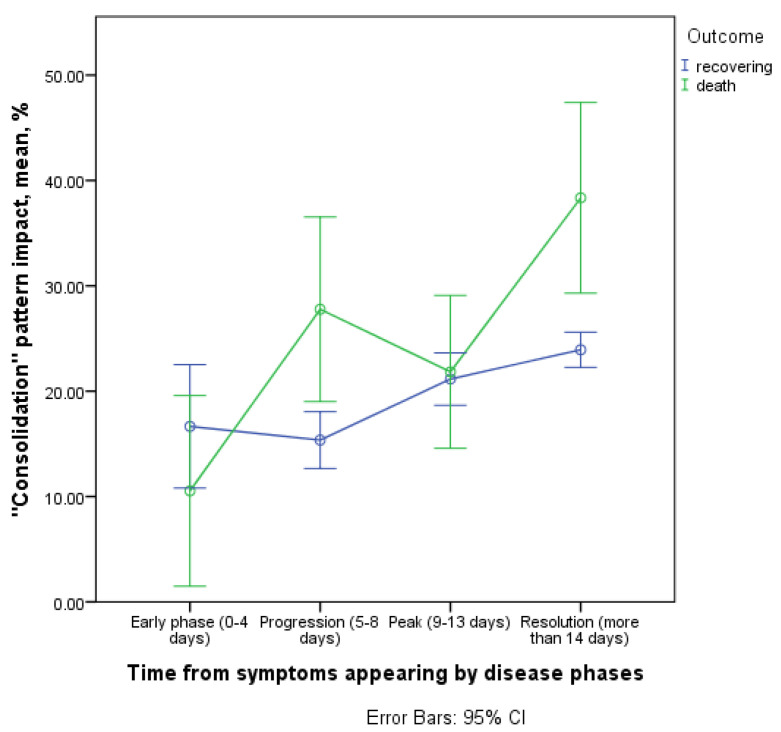
Contribution of the consolidation pattern to CT picture in the affected areas.

The contribution of consolidation pattern in lung damage picture showed an increase throughout disease in the group of deceased with a maximum rate between early phase (0–4 days) and progression phase (5–8 days) and between peak phase (9–13 days) and resolution phase (>14 days, Figure 12).

Among those who recovered, the contribution of the consolidation increased only starting from the sixth day of the disease and at a lower rate. Therefore, a statistically significant difference in the temporal dynamics was observed in groups with different outcomes of the disease (interaction of the time and outcome factor, *p* = 0.006). Significant differences were found between the recovered and deceased patients in the consolidation pattern contribution to total lung injury in the progression phase (15.4; 95% CI 12.2–18.5 vs. 27.8; 95% CI 18.8–36.7) and resolution phase (23.9; 95% CI 22.3–25.5 vs. 38.3; 95% CI 31.1–45.6), significance for the isolated outcome factor was *p* = 0.034.

#### 3.3.5. Contribution of Reticular Changes to CT Picture in the Affected Areas

**Figure 13:**
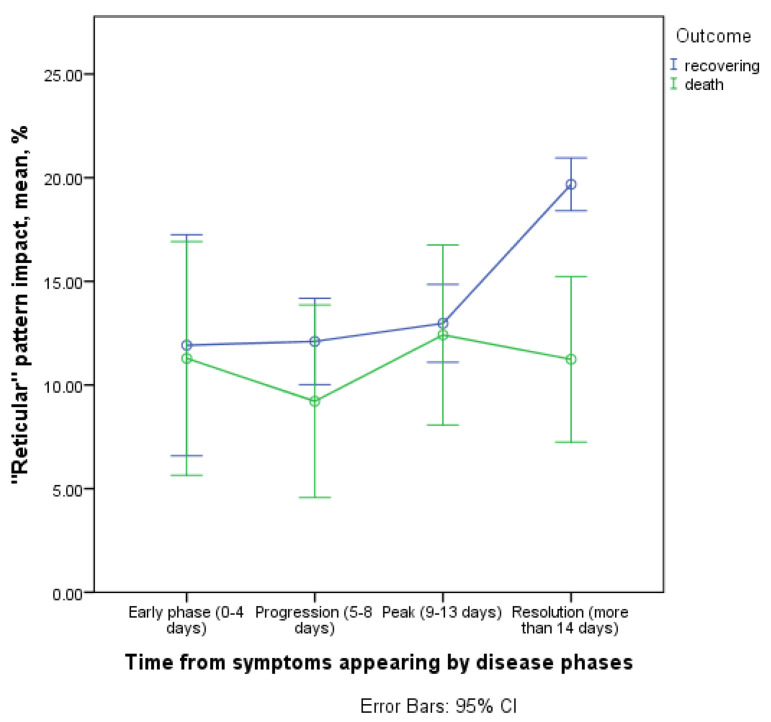
Contribution of reticular changes to CT picture in the affected areas.

There were no significant differences between the two cohorts of patients in the prevalence of RC and the dynamics of this pattern contribution (Figure 13).

These changes appear in the progression phase (5–8 days), followed by an increase in their contribution to the affected lung tissue in the group of recovering patients during the peak and the resolution phases. In the resolution phase in the group of deceased patients there was a significantly lower average value of the RC contribution to the lung damage picture, due to larger impact of the consolidation and CP patterns (11.2; 95% CI 5.8–16.7 vs. 19.7; 95% CI 18.5–20.9 in recovering patients). These findings indirectly demonstrate the role of RC as a potential marker of lung parenchyma reparation.

#### 3.3.6. Contribution of Linear Bands to CT Picture in the Affected Areas

**Figure 14:**
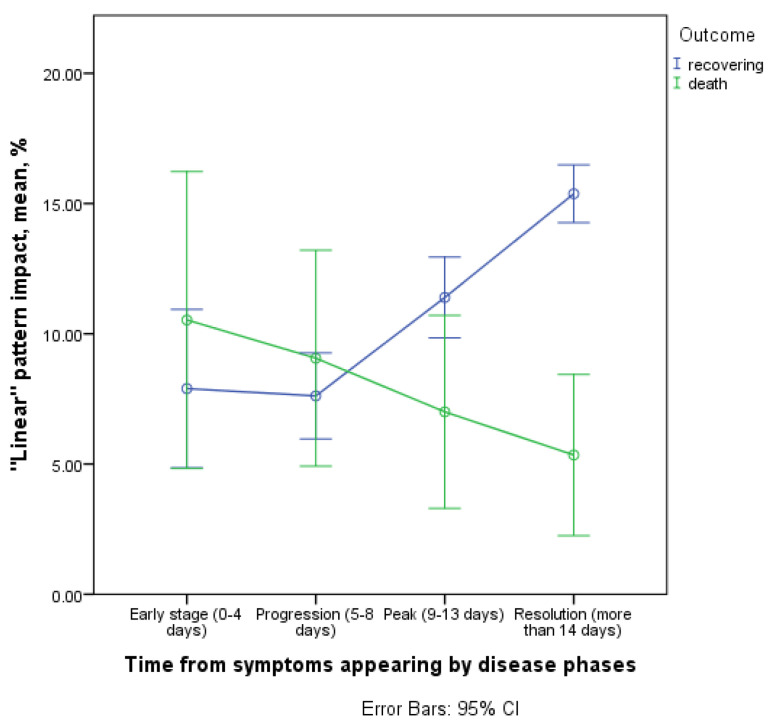
Contribution of linear bands to CT picture in the affected areas.

In the surviving and deceased patients, there were statistically significant differences in the linear bands’ contribution to the lung damage dynamics (interaction of the time and outcome factor, *p* = 0.005, Figure 14).

As of 8 days after symptom onset, the dynamics were different: in the cohort of survivors, there was an increase in the values of this pattern contribution with the course of the disease, while in deceased—on the contrary (mean values and CI in the resolution phase in survivors are 15.4; 95% CI 14.4–16.3, in decease—5.3; 95% CI 0.8–9.9). Based on these results, we can confirm the role of this CT pattern as one of the markers of reparative changes in the lung parenchyma.

#### 3.3.7. Dominant Patterns on Admission Day, Frequency of Pattern Detection

On admission day the dominant patterns of changes (according to the average contribution in the overall CT picture) in recovered patients were patterns of GGO (48.6%), CP (16.6%), consolidation (14.8%), RC (12%) and linear bands (8%), in deceased—35%, 23%, 20.5%, 12.5%, and 9%, respectively.

The frequency of the CP pattern detection in the cohort of deceased in all phases of the disease was higher than 60% of patients, reaching the maximum value (90.5%) in 0–3 days. The frequency of the consolidation pattern detection in the cohort of the deceased starting from the sixth day after symptom onset was 100%. At the same time, RC in both patients’ cohorts, starting from 3–6 days of the disease, registered in all disease phases in more than half of the cases, which also confirms the early activation and simultaneous (with damaging) course of the reparative process in the lung parenchyma.

## 4. Discussion

This research revealed several patterns reflecting typical CT changes and their evolution during the natural course of coronavirus infection in adult patients hospitalized in a repurposed hospital.

Our results of the occurrence of a particular CT sign of coronavirus infection reproduce the results of most of the aforementioned research papers and meta-analyses [32,33], except RC and CP. The overall frequency of CP registration during COVID-19 according to a meta-analysis is 20% of cases [32], the dispersion of the frequency according to different authors varies from 5% to 90% of cases, it may not be quite true due to the nature of statistical processing of data (for example, incorrect identification of the pattern, separate registration of its components—a thickening of the intra- and interlobular septa, and GGO). The total frequency of RC, according to some authors, is 10% [32], which is probably also due to the peculiarities of statistical data processing (thickening of intra- and interlobular septa are also components of the CP pattern).

GGO—according to our data, as well as according to the data of most authors—was the dominant type of changes in recovered patients at all phases, also one of the prevailing types of changes in deceased patients at all phases, which excludes its prognostic significance. In addition, the trend of the temporal dynamics of this type of change is identical to that of the lung damage severity. However, with the isolated detection of areas of GGO, it is possible to assume the presence of an early or resolution phase of the disease, which are described by other authors too [33,34,35,36,37,38].

Moreover, according to Shi et al., the presence of isolated GGO was noted at the subclinical stage of the disease (before the onset of symptoms) [34], and S. Inui et al. demonstrated that this is the dominant type of change in asymptomatic individuals [35]. It should also be noted that GGO is more common in patients younger than 50 years [44].

We also noted that consolidation is an indicator of the progressive phase of the disease, it develops, as a rule at the site of GGO while most often the GGO areas themselves progress in localization and size and coexist with consolidations [31,36]. Besides, it is more common in patients older than 60 years and people with a fatal outcome or in need of intensive care [15,31,42].

Contemporaneously, our study also revealed new patterns: we convincingly demonstrate a significant prevalence of changes in the type of CP in the cohort of deceased patients during CT examination at any time (*p* < 0.0005 at all stages of the disease), which confirms the prognostic value of this pattern. Similar results were published in only one article by Feng Pan and co-authors [45].

Other authors observed CP more often in the resolution stage of the disease and in people over 60 years old. Probably, the point of that ‘mosaic’ pulmonary parenchyma in CP detected in recovering patients is due to reparative changes and the appearance of areas of normal/increased airiness against the background of disturbed areas, while in deceased patients it is most likely associated with worsening damage to the previously airiness pulmonary parenchyma (joining of the interstitial component to the alveolar, or vice versa).

Most authors agree that the detection of CP in combination with ‘GGO’ and (or) consolidation corresponds to the peak phase of the disease [31,42,46].

We confirm that CP often co-exists with the patterns of pulmonary parenchyma density increasing (GGO and consolidations) [36]. Perhaps this is due to the fact that the CP is a pattern reflecting the damage of both the alveolar and interstitial components of the pulmonary parenchyma [39], not just one as in GGO and not to the same extent as in consolidation. At the same time, the histological basis of the ‘CP’ pattern and the reticular pattern are the same types of changes, but of different severity [33].

According to Feng Pan and co-authors, the areas of GGO were caused by intra-alveolar edema or protein exudate, but according to Pershina et al. [47], an increase in the number of cells, hyaline membranes, and desquamation of the alveolar epithelium was detected in the lumen of the alveoli at autopsy of more seriously ill patients. In any case, both types of changes lead to a decrease in the area of the functional surface of the lung involved in gas exchange. We can only assume that in the case of exudative edema, the reparative processes are faster and more effective

The data of Feng et al. and Pershina et al. show that the histologic substrate of the interstitial pattern is edema and lymphocytic infiltration of the interlobular and intralobular septa or deposits of collagen in them. The latter is also a symptom of later-phase DAD and probably impairs drainage of the lymph and venous blood, and also reduces the efficiency of gas diffusion, providing a mutually aggravating relationship effect.

Histological substrate underlying CT-patterns, according to different studies focusing on the analysis of data from autopsy patients from COVID-19 can be three different forms: (1) diffuse alveolar damage (DAD) is usually a nonspecific response of the lung parenchyma to a multitude of injurious agents, and according to Brogna et al., aforementioned findings may reflect different phases of this form [48]; (2) organizing pneumonia—active (periodically aberrant) regeneration of lung parenchyma; and (3) acute fibrinous and organizing pneumonia (AFOP) [49,50,51]. Some authors suggest that these patterns may relate to the spectrum of manifestations of damage and repair processes in pulmonary parenchyma at different stages. For example, the AFOP pattern was revealed in the works devoted to the patients autopsy on the 20th day of the disease [52], the exudative and fibrotic phases of DAD were revealed on the early or, conversely, late (on the 32nd day) phases, respectively [53]. The coexistence of these patterns is often diagnosed, with the presence (more often in young people) of OP-type changes [50,51].

The main findings in DAD are capillary congestion, interstitial and intraalveolar edema associated with the fibrin deposition (fibromyxoid exudate), and hyaline membrane formation [54]. Hyaline thrombi in the microvascular compartment may appear due to coagulation mechanisms violations and endothelial damage [40], signs of fibroblast proliferation may appear, reactive hyperplasia of second-type pneumocytes with desquamation or atypia (large nuclei) may occur later [55].

OP is manifested by the formation of granulation tissue and polypoid plugs that fill the lumen of the alveolar ducts and terminal bronchioles (Masson’s body). The pattern is sensitive to corticosteroid therapy [56].

A feature of AFOP is the presence of signs inherent in DAD and OP, with a unique feature—extensive filling of the alveolar lumen with “fibrin balls”, and not hyaline membranes, with fibroblast bodies and fibroblasts surrounding these “balls” [51,57].

Severe patients were also less characterized by changes in the GGO type in primary lung CT (*p* = 0.007), this was due to a change in the balance towards CP and consolidation. In our opinion, this may indicate a faster dynamic of the process in this group of patients.

RC may be the result of “healing” (or the stage of proliferation of the inflammatory process with the gradual replacement of the once functional parenchyma by connective tissue) [42]. It often co-exists with the patterns of pulmonary parenchyma density increasing (GGO and consolidations) [36] as injuring and reparative processes in lung tissue are parallel. With a prolonged course of the disease, this pattern can progress and prevail [33], perhaps due to very slow resolution of these changes.

**Figure 15:**
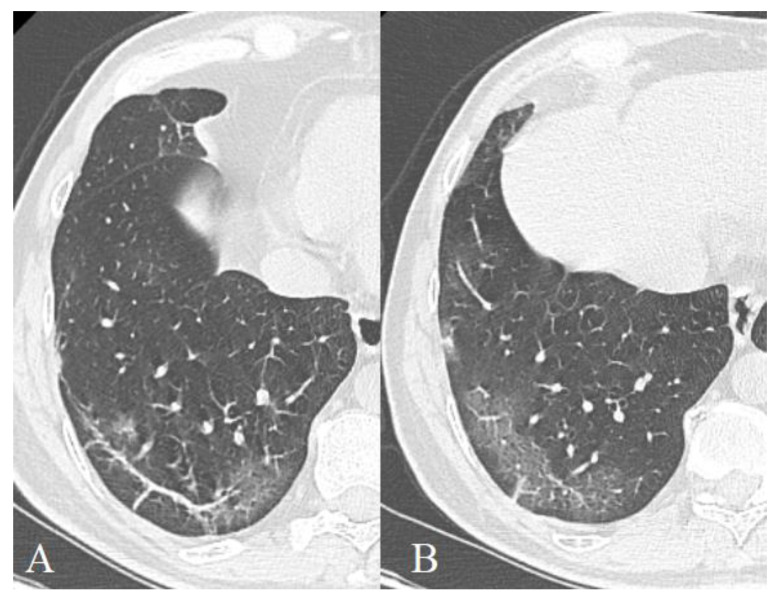
56-year-old patient, subpleural linear bands (**A**) transform to focal ground-glass opacities (**B**).

However, we discovered that—in the later phases of the disease—linear strands were less characteristic for the group of severe patients (*p* = 0.003), while the frequency of RC did not significantly differ (*p* = 0.018), which in our opinion may be due to the greater sensitivity of the pattern linear strands as a marker of repair, compared with the pattern of RC. This indicates that processes of repair in deceased patients are less pronounced compared to the processes of damage to the pulmonary parenchyma. In addition, the appearance of such bands may be due not only to a violation of air patency in a separate pulmonary lobule but also to an increase in perfusion of gravity-dependent areas of the lung against the background of persistent edema and exudate, which probably did not occur in deceased patients [58]. Additionally, most of those bands are not fibrosis (as was declared in the first papers) but little linear atelectasis (Figure 15).

Given the statistically significant difference between the recovered and died in the average values and CI gap of CP pattern contribution in the early and progression phases, and consolidation pattern contribution in the progression and resolution phases, it is possible to assume gradual worsening of the lung parenchyma damage while maintaining the same lesion volume change trend as in recovered patients

There has been an increasing CP contribution in recovered patients (between the early and progression phases) and in the deceased (between progression and peak phases): the gap between the average values and CI in these cohorts during the peak phase is 15.4; 95% CI 13.3–17.5 and 28.7; 95% CI 22.5 to 34.8, respectively. This finding suggests that in the latter cohort of patients there is more extended in time progression phase, in comparison with recovered patients, which probably leads to the deterioration of the prognosis. This may be due to the long-term persistence of the virus in the lung tissue, which is probably a trigger for repeated lung damage and disease progression [59].

According to our data, the prevalence of the CP pattern is also noted in the last CT scan of deceased patients and consolidation patterns are more pronounced (*p* = 0.004). This may be due to different phases of the disease at the time of the last CT scan among the surviving and deceased patients; indirectly, it confirms some authors observation that depending on pulmonary parenchyma lesion severity at the beginning of the process, the time frame of the phases described above may shift. In addition, the process dynamics as showed by Yu et al. seem to be different in cohorts of patients who differ in the process severity [60]. For example, in severe and extremely severe cases, the peak phase may occur later than the 15th day, and in asymptomatic individuals or patients with a mild degree of the disease, it occurs on the 3rd day from the moment of the development of clinical symptoms [61].

We also reproduced data of most authors according to the types of lung tissue changes severity, localization, shapes, and contours in recovered and deceased patients.

According to our data in all phases of the disease, the scale of destruction of the lung parenchyma, estimated by the sum of points of the affected segments was more pronounced in severe patients (all *p* < 0.0005).

The works of many authors have also demonstrated the role of lesion volume as an independent risk factor for increased mortality in COVID-19 [62,63,64]. Thus, the study by Ruch et al. showed a significantly higher frequency of deaths (70%) in individuals with more than 50% of the pulmonary parenchyma involved in the process [65]. In addition, patients with severe and extremely severe variants of the course also had significantly higher scores obtained when evaluating the severity of pulmonary parenchyma lesions [66,67,68,69,70].

According to some authors, bilateral lesions mainly in the basal and central parts [71], or diffuse [45] were more common among deceased patients, which was observed by us as well and is quite natural given the increase in severity with the involvement of more and more areas of the pulmonary parenchyma.

In recovered patients, we observed the peripheral location of pathological changes predominantly (in 70.5% of cases against 40% of cases in deceased patients).

The “tropism” of pathologic changes to the peripheral and posterior parts of the lungs (which we reported above) can be explained by a more numerous and wide representation of blood and lymphatic vessels, as well as bronchioles in these parts [72].

At the same time, there was no significant difference in the volume of damage to the right and left lungs in concordance with our data, in contradiction to reports by other authors.

The dynamics of changes in the damage scale and localization revealed by us during the disease course generally corresponds to those described by other authors [42].

Taking into account the presence of residual (not only ‘reparative’) changes on CT examinations of the lungs upon discharge from the hospital in most patients, in our opinion, further studies are also needed to assess the long-term consequences of the coronavirus infection.

With regard to the clinical results of our study, it should be noted that the risk factors in our population are similar to those previously reported in other articles and reviews, with the exception of arterial hypertension. We have discussed in detail the possible reasons for this in another article on risk factors in our patient cohort [27]

## 5. Limitations of Our Study

It should be noted that our study had several limitations that could influence the results to some extent:

1. Some combinations of radiological patterns were in the ‘gray’ zone, which makes it possible that certain patterns were not registered correctly, as in many other analyzed articles.
2. The volume of lung lesion was registered visually, according to scales that are not devoid of shortcomings and subjectivity.
3. The sample of fatal cases was relatively small, which reduced the sensitivity of the statistical analysis.
4. This study does not include data from pathomorphological studies, which makes our arguments about the mechanisms of development of certain patterns purely hypothetical.
5. There may have been a hidden mutual influence of factors (confounding), which may distort the results of our observational study.

## 6. Conclusions

Thus, our research on a large enough sample of symptomatic patients hospitalized in a repurposed hospital reproduced data on typical pulmonological CT manifestations of infection, their frequency of occurrence, and the prognostic significance of the scale of the lesion. In addition, when comparing two cohorts of patients (recovered and deceased), new patterns were found and differences in the dynamics of the process were investigated: the CP pattern, which is more common and has a greater contribution to the overall CT picture in different phases of the disease in deceased patients, has isolated prognostic significance and is probably a reflection of faster dynamics of the process with a long phase of progression of pulmonary parenchyma damage with an identical trend of changes in the scale of the lesion (as recovered) in this group of patients. The revealed patterns certainly require further research and can be reflected in clinical practice.

## Figures and Tables

**Table 1:** Summary of key epidemiological risk factors findings from the previously published article of our colleagues [27].

Risk Factor		N_R_	N_D_	OR
Gender (m vs. f)	Male	314	37	1.97 * (95% CI 1.07–3.62)
	Female	268	16	
Gender (m vs. f, aged 59 or more)	Male	125	25	1.83 (95% CI 0.92–3.62)
	Female	137	15	
Gender (m vs. f, under 59 years old)	Male	189	12	8.32 * (95% CI 1.07–64.75)
	Female	131	1	
Body mass index	<25	124	11	0.78 (95% CI 0.38–1.60)
	≥25	420	29	
Body mass index	<30	323	27	0.70 (95% CI 0.36–1.39)
	≥30	221	13	
Current smoking	Yes	51	5	1.17 (95% CI 0.44–3.08)
	No	525	44	
Alcohol abuse (self-reported)	Yes	5	3	7.13 * (95% CI 1.65–30.72)
	No	570	48	

N_R_—number of patients who had the risk factor in the recovered group; N_D_—number of deceased patients who had the risk factor, OR—odds ratio. OR marked with an asterisk (*) are statistically significant.

**Table 2:** Summary of occurrence frequency of CT patterns depending on the disease radiology phase according to our data.

CT-Pattern	Early Phase		Progressive Phase		Peak Phase		Resolution Phase	
	%_R_	%_D_	%_R_	%_D_	%_R_	%_D_	%_R_	%_D_
Ground glass opacity	81.8	94.4	93.1	87.0	87.3	83.3	86.1	83.8
Crazy paving	38.7	66.7	56.0	72.7	50.2	86.7	36.3	61.8
Consolidations	48.0	47.4	56.9	87.0	67.3	76.7	71.6	91.7
Reticular interstitial changes	36.5	55.6	53.0	47.8	53.8	64.5	71.1	61.1

_R_—recovered patients; _D_—deceased patients.

## Data Availability

The datasets generated during and/or analyzed during the current study are available from the corresponding author on reasonable request.
